# Effects of embryonic thermal manipulation on the immune response to post-hatch *Escherichia coli* challenge in broiler chicken

**DOI:** 10.14202/vetworld.2023.918-928

**Published:** 2023-05-07

**Authors:** Mohammad Borhan Al-Zghoul, Ziad Waheed Jaradat, Mustafa M. Ababneh, Mohammad Ziad Okour, Khaled Musa Mohammad Saleh, Ayesha Alkofahi, Mohammad Hussien Alboom

**Affiliations:** 1 Department of Basic Medical Veterinary Sciences, Faculty of Veterinary Medicine, Jordan University of Science and Technology, Irbid, Jordan; 2 Department of Biotechnology and Genetic Engineering, Faculty of Science and Art, Jordan University of Science and Technology, Irbid, Jordan; 3 Department of Biology and Biotechnology, Faculty of Science, The Hashemite University, Zarqa, Jordan

**Keywords:** broiler, challenge, *Escherichia coli*, immune response, incubation, thermal manipulation

## Abstract

**Background and Aim:**

Thermal manipulation (TM), exposure to mild heat shock during embryogenesis, which is a critical developmental period of broiler chickens, improves tissue stability, oxidative stress response, and immune response during heat stress. Thermal manipulation could be more cost-effective than other methods to boost the immune response. This study aimed to evaluate the impact of TM during embryogenesis, concomitant with an *Escherichia coli* challenge, on body weight (BW), body temperature (T^b^), and splenic mRNA expression of cytokines (Interleukin [IL]-1β, IL-2, IL-6, IL-8, IL-12, IL-15, IL-16, IL-18, and interferon [IFN]-γ) in poultry.

**Materials and Methods:**

A total of 740 fertile eggs were procured from a certified Ross broiler breeder. The eggs were divided into two incubation groups: the control and TM groups. The eggs in the control group were kept at 37.8°C air temperature and 56% relative humidity (RH) during incubation; eggs of the TM group were incubated under standard conditions, except for embryonic days 10–18, during which they were incubated at 39°C and 65% RH for 18 h daily. On the 7^th^ day of incubation, eggs with dead embryos were excluded. After hatching was complete, each group was further subdivided into saline-treated or *E. coli*-challenged groups. The *E. coli* (serotype 078 with the dose of 1.5 × 10^5^ colony-forming unit/mL) challenge was performed when the birds were 20 days old. Body weight and T^b^ measurements were taken on post-hatch days 20, 21, 23, and 25. Splenic mRNA expression of cytokines (IL-1β, IL-2, IL-6, IL-8, IL-12, IL-15, IL-16, IL-18, and IFN-γ) was analyzed by real-time quantitative polymerase chain reaction.

**Results:**

Following the *E. coli* challenge, the TM-treated group’s body performance parameters (BW and T^b^) were significantly increased compared with the control group. Body weight was higher in the TM group than in the control group (p < 0.05); T^b^ was lower in the TM group than in the control group (p < 0.05). The mRNA levels of IL and IFN-γ were more stable and moderately induced in the TM group compared with the control group. Thermal manipulation altered the basal mRNA levels of ILs and IFN-γ and changed their expression dynamics after the *E. coli* challenge.

**Conclusion:**

Thermal manipulation during embryogenesis could boost the immune system response to *E. coli*.

## Introduction

Bacterial infection is a significant problem in the poultry industry [1] and is a severe hazard to broiler chickens, resulting in considerable economic losses [2]. *Escherichia coli*, a facultative anaerobic Gram-negative, non-acid-fast, and non-spore-forming bacillus, is present in the intestine of poultry [3]. Extraintestinal pathogenic *E. coli* strains cause extraintestinal illnesses [4, 5]. Because bacterial infections are stressful for poultry producers, it is necessary to boost the immune system of poultry by administering antibiotics [6]. Unfortunately, the extensive use of antibiotics as treatment or prophylactic has led to bacterial antibiotic resistance in zoonotic enteropathogens (e.g., *Salmonella* and *Campylobacter*) and commensal bacteria (e.g., *E. coli* and *enterococci*) [7].

Therefore, studies have focused on using nutritional supplements such as omega-3 polyunsaturated fatty acids, vitamin E [8], phosphorus [9], fish oil [10], *Bacillus subtilis* [11], medicinal mushrooms [12], calcium [13], commercial organic acid [14], crude extract of *Peganum harmala* [15], and amla extract [16], to boost the immune system of poultry against bacterial infections [12]. Furthermore, bacterial vaccines are used to protect broilers from bacterial infections [17], such as autogenous *E. coli* vaccine [18], aroA-deleted vaccine [19], live *E. coli* vaccine [20], and bacteriophage aerosol spray [21]. Unfortunately, most of these methods are costly and difficult to use.

Thermal manipulation (TM) or exposure to mild heat stress during embryogenesis, which is critical for broiler chicken development, has been shown to improve tissue stability [22], oxidative stress response [23], and immune response during heat stress [24, 25]. Thermal manipulation is suggested as an alternative method to improve the response of poultry to heat stress [22, 23, 26]. Studies have shown that TM can improve broiler hatchability [27], BW [28], performance, meat quality [29], thermotolerance acquisition [27, 30, 31], and overall positive immune response to heat stress [32]. Thermal manipulation has been shown to increase intestinal integrity and mucus production and reduce the stress response in broiler chickens against post-hatch *Salmonella enterica* Enteritidis inoculation [29]. Shanmugasundaram *et al*. [33] reported that TM of Pekin duckling embryos significantly decreased the levels of bursal and splenic interleukin (IL)-6 mRNA and heat-shock protein 70 (Hsp70) following lipopolysaccharide (LPS) challenge.

Thermal manipulation, the modification of the temperature and humidity of incubation of avian eggs, is applied during embryogenesis, in a cyclic period, and at specific time intervals of embryogenesis [34]. It is cost-effective and simple, and several studies have confirmed its efficacy [35–37]. Thermal manipulation was linked to changes in the basal and dynamic expression levels of essential signaling proteins important for tissue integrity and repair, which contributed to the improved development of thermotolerance and immune response during heat stress [22, 26]. A positive impact of TM on body temperature (T^b^), body weight (BW), and the immune response in post-hatch broiler chickens have been reported [34, 38–41].

Thermal manipulation has been reported to improve the immune response of broiler chickens during acute and chronic heat stress [25, 42]. Thermal manipulation can modulate the expression levels of splenic mRNA cytokines (IL-1β, IL-4, IL-6, IL-8, IL15–18, interferon [IFN]-α, IFN-β, IFN-γ, and tumor necrosis factor-α) and genes involved in cytokine induction pathways in broiler chicken embryos [42]. Most of these IFNs and ILs have a role in combating bacterial infection [43]. Therefore, if TM could improve the immune response to heat stress, it may also improve the immunological response to bacterial infection. Shanmugasundaram *et al*. [33] suggested that TM during the incubation of Pekin duckling embryos may stimulate the immune system, which may be beneficial for subsequent post-hatch inflammatory challenges.

It has been found that higher incubation temperatures during mid-incubation and post-hatch early feeding resulted in reduced immune function and an increased risk of infection, including colibacillosis [44]. By contrast, lower incubation temperatures during late incubation and delayed early feeding strategy were associated with impaired broiler chicken resilience to necrotic enteritis in later life [45]. However, the effects of TM during broiler chicken embryogenesis on immune system parameters during a post-hatch bacterial challenge have not been reported.

This study evaluated the impact of TM during embryogenesis and subsequent bacterial infection on the parameters of immune system function.

## Materials and Methods

### Ethical approval

All experiment management conditions and procedures employed in this study were approved by the Animal Care and Use Committee of Jordan University of Science and Technology (JUST) (approval #: 475/2020).

### Study period and location

The study was conducted from 5 June 2020 to 25 August 2021 at the Animal House Unit (Faculty of Veterinary Medicine, Jordan University of Science and Technology University, Irbid, Jordan).

### Study population and incubation

A total of 740 hatching eggs from a Ross broiler breeder (36 weeks old) were obtained from certified distributors in Irbid, Jordan. The obtained eggs were examined for any damage. Abnormal eggs, very light (<55 g), and very heavy (>70 g) eggs were excluded from the study. The selected eggs (average weight 63 ± 2 g) were incubated in four commercial Type-I HS-SF incubators (Masalles, Barcelona, Spain). The eggs were divided into two incubation groups: the control and TM groups. Eggs in the control group were incubated at 37.8°C air temperature and 56% relative humidity (RH) throughout embryogenesis. By contrast, eggs in the TM group were incubated under standard conditions, except on embryonic day (ED) 10–18, during which they were incubated at 39°C air temperature and 65% RH for 18 h daily. Hatching eggs were incubated from day 1 under controlled conditions (37.9°C, 56% humidity, 12-h light–dark cycle, and automatic hourly rotation). On the 7^th^ day, the eggs were examined by candling: Infertile eggs and eggs with dead embryos were excluded from the study.

### Hatching management and rearing

On hatching day, hatched chicks were counted every hour. After feather drying was complete, 1-day-old chicks were transported to the Animal House Unit at JUST, where the field experiment was conducted. Room temperature was maintained at 33°C ± 1°C during the 1^st^ week and was progressively dropped to 24°C by the end of the 3^rd^ week. From day 24 after hatching until day 35, the temperature was kept at 21°C. Standard feed rations and water were provided to the chickens *ad libitum* during the field experiment. At the end of the experiment, on day 35 of age, all the broiler chickens were humanely euthanized.

### Inoculation

The *E. coli* serotype 078 used for inoculum preparation was provided by the microbiology laboratory at JUST. The bacterial strain was cultured on Muller–Hinton broth at 37°C overnight with shaking. Two milliliters of the culture were centrifuged at 5000× *g* for 5 min at 4°C. The pellet was washed twice with 2 mL of 1× sterile phosphate buffer saline, resuspended completely in the buffer, and centrifuged at 5000× *g* for 5 min at 4°C. The pellet was then resuspended in 2 mL of 0.9% normal saline (NS). The suspension’s optical density was adjusted using a spectrophotometer, and dilutions were made in NS to obtain the desired concentration.

### Bacterial challenge

The *E. coli* challenge was performed when the birds were 20 d old. Both groups of broilers (control and TM) were further subdivided into two groups: A NS subgroup (n = 100 each), which was injected intraperitoneally with 0.5 mL of 0.9% NS, and an *E. coli* subgroup (n = 100 each), which was injected intraperitoneally with 0.5 mL of *E. coli* (1.5 × 10^5^ colony-forming unit/mL). Then, broiler chickens were transported to the experiment room and were kept in thermoneutral conditions (24°C ± 1.0°C). The mortality rate during this stage was calculated. At pre-injection (day 0) and post-injection days 1, 3, and 5 before bleeding, BW and T^b^ were recorded for randomly selected broilers using a J/K/T thermocouple meter equipped with a rat rectal probe (Kent Scientific Corp., CT, USA; ±0.1°C).

### RNA isolation and cDNA synthesis

On post-injection days 1, 3, and 5 of *E. coli* or NS, 40 broiler samples were collected from all treatment groups per day. Spleen tissues were carefully collected and snap-frozen on-site using liquid nitrogen to prevent RNA degradation. Samples were then kept in TRI Reagent solution tubes (Zymo Research Co., CA., USA) and stored at −20°C. Tissues were homogenized using Bead Ruptor Elite-Bead Mill Homogenizer (OMNI International, Kennesaw, GA, USA). Total RNA was isolated from splenic samples using Direct-Zol RNA MiniPrep (Zymo Research Co.) with TRI Reagent (Zymo Research Co.). The quantity and quality of the RNA was evaluated using a Qubit 4 Fluorometer (Thermo Fisher Scientific, MA, USA) and a Biotek PowerWave XS2 Spectrophotometer (BioTek Instruments, Inc., Winooski, VT, USA) and 1% agarose gel, respectively. cDNA was synthesized for each sample using a PrimeScript RT Master Mix (Zymo Research Co.), using 500 ng of RNA for each reaction.

### Real-time quantitative polymerase chain reaction (qPCR)

Blastaq Green qPCR Master Mix (Applied Biological Materials Inc., Richmond, Canada) was used in a Rotor-Gene Q MDx 5 plex instrument (Qiagen, Hilden, Germany). Briefly, a 20 μL reaction mix was prepared from 10 μL of the master mix; 2 μL forward primer (2 pmol); 2 μL reverse primer (2 pmol); 2 μL cDNA of the sample; and 4 μL of nuclease-free water. Cycling parameters were 50°C for 2 min, 95°C for 15 min, and 40 cycles of 95°C for 10 s, followed by 30 s at 57°C and 72°C for 10 s, with final melting at 95°C for 20 s. Duplicates from each cDNA were analyzed, fluorescence emission was detected, and relative quantification was performed automatically. β-Actin, 28R sRNA, and glyceraldehyde-3-phosphate dehydrogenase were used as internal controls to which the fold changes in gene expression were normalized. The melting curve approved the specificity of single target amplification.

The cDNA sequence for each gene was obtained from NCBI’s Nucleotide database (https://www.ncbi.nlm.nih.gov/nucleotide/). The IDT Primer Quest software (http://eu.idtdna.com/PrimerQuest/Home/Index) was used to create all primers. The primer sequences are presented in Table-1.

**Table-1:** Primer sequences that are used in the real-time qPCR analysis.

The gene	Sequence (5’-3’)	Annealing temperature (°C)
28S rRNA	F: CCTGAATCCCGAGGTTAACTATT R: GAGGTGCGGCTTATCATCTATC	60
β-Actin	F: ACCGCAAATGCTTCTAAACC R: ATAAAGCCATGCCAATCTCG	60
GAPDH	F: TTGTCTCCTGTGACTTCAATGGTG R: ACGGTTGCTGTATCCAAACTCAT	60
IL-1 β	F: CCCGCCTTCCGCTACA R: CACGAAGCACTTCTGGTTGATG	60
IL-2	F: GAGAGCATCCGGATAGTGAAT R: TGTGGAGGCTTTGCATAAGAG	60
IL-6	F: AAATCCCTCCTCGCCAATCT R: CCCTCACGGTCTTCTCCATAAA	60
IL-8	F: CTTCCACCTTCCACATCGGT R: CATTTCCCCTAGCAAGCCCT	60
IL-12	F: CTGTGGCTCGCACTGATAAA R: CAATGACCTCCAGGAACATCTC	60
IL-15	F: TAGGAAGCATGATGTACGGAACAT R: TTTTTGCTGTTGTGGAATTCAACT	60
IL-16	F: TGAACCACAGGTGTCTGAGC R: TCAGCTTCTGGGCTTTACGG	60
IL-18	F: GATGAGCTGGAATGCGATGC R: TGGACGAACCACAAGCAACT	60
IFN-γ	F: ACCTTCCTGATGGCGTGAAG R: GCGCTGGATTCTCAAGTCGT	60

IFN=Interferon, IL=Interleukin, qPCR=Quantitative polymerase chain reaction, GAPDH=Glyceraldehyde-3-Phosphate Dehydrogenase

### Statistical analysis

All statistical analyses were conducted using IBM Statistical Package for the Social Science Statistics 26.0 (IBM software, Chicago, IL, USA). The hatchability rate was analyzed using a Chi-square test. Body temperature, BW, and serum levels of cytokines and expression of mRNAs were expressed as means ± standard deviation. For each experimental time point of T^b^ and BW, an independent t-test was used to compare mean parameters in control versus TM. Two-way analysis of variance was also used to compare T^b^, BW, and mRNA expression-level changes within the treatment groups (control vs. TM) but at different time intervals after NS *E. coli* injections (day 0 vs. days 1, 3, and 5). Parametric differences were considered statistically significant at p < 0.05.

## Results and Discussion

This study aimed to examine the impact of TM and *E. coli* challenge on BW and T^b^, as well as splenic mRNA levels of immune response genes (IL-1β, IL-2, IL-6, IL-8, IL-12, IL-15, IL-16, IL-18, and IFN-γ), in post-hatch broiler chickens. The TM-treated group was exposed to 39°C for 18 h and 65% RH during ED 10–18.

### Effects of TM and *E. coli* challenge on BW and T^b^

**Figure-1:**
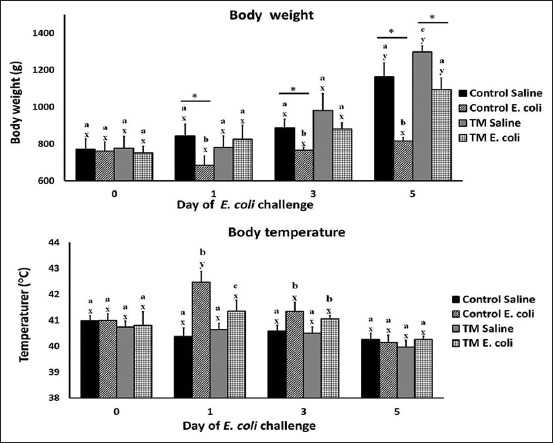
Effects of *Escherichia coli* challenge on body weights and body temperatures in broiler chickens subjected to thermal manipulation (TM) during embryogenesis (n = 100). *,* Within the same day, the mean of TM group is significantly different compared to the mean of the control (p < 0.05). ^a-b^Within the same day and between different treatment groups, means with different superscripts is significantly different (p < 0.05). ^w-z^Within the same treatment group and between day 0 versus. days 1, 3, 5, and 7 after *E. coli* challenge, means with different superscripts are significantly different (p < 0.05).

Figure-1 depicts the effects of TM and *E. coli* challenge on BW and T^b^ of broiler chickens. On day 0 before *E. coli* challenge, all tested groups had comparable BW. After days 1, 3, and 5 of saline injection, a slight increase was recorded in the BW of the control group on day 1, and a significant increase was recorded on days 3 and 5 compared with that on day 0. Similarly, in the saline-injected TM group, a slight increase in BW was recorded on day 1 and significant increase on days 3 and 5 compared with that on day 0 and controls (p < 0.05). Interestingly, in the *E. coli-*challenged control group, a significant decrease in BW was recorded on days 1, 3, and 5 post-*E. coli* challenge compared with that on day 0 and saline-injected groups on similar days (p > 0.05). However, in the TM group, a slight reduction in BW was recorded on day 1 (p > 0.05), and a significant reduction of BW on days 3 and 5 post-*E. coli* challenge compared with that on day 0 and with saline-injected groups on similar days. Interestingly, higher reductions in BW in *E. coli-*challenged groups were observed in control compared with the TM group (p < 0.05).

*Escherichia coli*-infected broiler chickens usually exhibit a remarkable decrease in BW [46], which can result from appetite loss [43]. In this study, *E. coli*-challenged groups recorded lower BW on days 1, 3, and 5 compared with saline-injected groups. The *E. coli*-challenged control group recorded a higher reduction in BW than the TM *E. coli*-challenged group, indicating a higher resistance of the TM group to *E. coli* infection and a lower level of symptoms associated with infection. This study is the first to investigate the effect of TM and *E. coli* challenge on the BW of broiler chickens. However, the previous findings can explain the higher reduction in BW in the *E. coli*-challenged control group compared with the TM *E. coli*-challenged group, because TM was reported to improve physiological characteristics, such as the immune response [25], tissue stability [32], and oxidative stress tolerance [23]. Indeed, these characteristics can positively impact the chicken’s ability to resist *E. coli* infection and eliminate manifestations of such infections, including loss of appetite and weight [47].

On day 0 before *E. coli* challenge, all tested groups had the same T^b^. However, in the TM and control groups, after days 1, 3, and 5 of saline injection, a slight decrease was recorded in T^b^. Interestingly, in the *E. coli-*challenged control group, a significant increase in T^b^ was recorded on days 1 and 3 compared with that on day 0 and with saline-injected groups on similar days (p < 0.05). In addition, in the TM group, a significant increase in T^b^ was recorded on days 1 and 3 post-*E. coli* challenge compared with that on day 0 and with saline-injected groups on similar days (p < 0.05).

In both groups, T^b^ was significantly lower on day 5 of post-*E. coli* challenge than that on day 0 (p < 0.05). In addition, the control group showed greater increases in the T^b^ *E. coli* challenge groups than the TM group (p < 0.05) (Figure-1).

*Escherichia coli*-infected broiler chickens usually represent a remarkable inflammatory response associated with increased T^b^ [48]. In this study, the *E. coli*-challenged control group recorded higher T^b^ than the TM *E. coli*-challenged group. The lower T^b^ in the TM group indicates an improved immune response against infection.

Saleh and Al-Zghoul [25] reported that TM could improve the immune response because a higher inflammatory response was seen in the control group compared with the TM group after heat stress. Tang *et al*. [49] investigated the effects of *E. coli* infection in broiler chickens exposed to heat stress and the *E. coli*-infected control group. Their results indicated that heat stress could improve the inflammatory response in broiler chickens and make them more resistant to *E. coli* infection. The intestines of heat-stressed broiler chickens were longer and heavier, with more *E. coli* in the cecum, than control chickens, and the small intestines of heat-stressed broiler chickens had a modified protective morphology at specific locations [49]. Al-Zghoul *et al*. [23] reported that TM improved thermotolerance in the long-term by improving the levels of mRNA and the total capacity of antioxidant genes related to heat-induced oxidative stress. Saleh and Al-Zghoul [25] reported that TM might increase thermotolerance by improving signaling proteins’ expression levels in maintaining tissue stability and mediating tissue repair after heat stress.

### Effects of TM and *E. coli* challenge on splenic IL-1β, IL-2, IL-6, and IL-8 mRNA expression

Interleukins are cytokines that are secreted from macrophages on infection. Interleukins work as immune polypeptides that activate immune cells and regulate proinflammatory responses [43, 50]. During *E. coli* infection, macrophages get activated by interacting with molecules on the surface of *E. coli* and overexpress proinflammatory cytokines, such as IL-1β [43]. Sun *et al*. [51] reported that during *E. coli* infection, monocyte production increases, which then differentiates into cytokine-producing macrophages. In this study, the mRNA levels of IL-1β decreased in TM *E. coli*-challenged compared with the control group at the same time intervals. This indicates the effect of TM in improving immune responses, including the dynamics of specific cytokines [25, 42].

**Figure-2:**
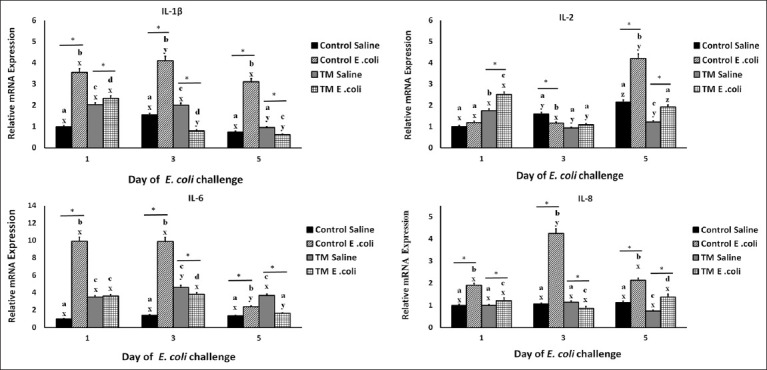
Effects of *Escherichia coli* challenge on the splenic mRNA level of interleukin (IL)-1β, IL-2, IL-6, and IL-8 in broiler chickens subjected to thermal manipulation (TM) during embryogenesis (n = 100). The ILs’ mRNA level of control Day-O (saline-injected) is set at 1-fold change, and the levels are presented as fold inductions relative to the control Day-O (saline-injected) group. *Within the same day, the mean of TM group is significantly different compared to the mean of the control (p < 0.05). ^a-b^Within the same day and between different treatment groups, means with different superscripts is significantly different (p < 0.05). ^w-z^Within the same treatment group and between day 0 versus. Days 1, 3, 5, and 7 after *E. coli* challenge, means with different superscripts are significantly different (p < 0.05).

In the saline-injected control and TM groups, a higher expression of IL-1β was detected in the TM group compared with the control on days 1 and 3 (p < 0.05). However, after *E. coli* challenge on days 1, 3, and 5, a remarkable increase in the levels of IL-1β mRNA was observed in the control groups compared with the control saline-injected and TM *E. coli*-challenged groups (p < 0.05). Interestingly, in the TM *E. coli*-challenged group, an increase in IL-1β mRNA was observed only on day 1; however, on days 3 and 5, the levels of IL-1β mRNA decreased compared with the control group at the same time intervals (Figure-2) (p < 0.05).

In the saline-injected control and TM groups, higher expression of IL-2 was detected in the TM group compared with the control group on day 1 (p < 0.05). However, on days 3 and 5, the control group reported higher IL-2 expression compared with the TM group. After the *E. coli* challenge, a remarkable increase in IL-2 expression was detected in the TM *E. coli-*challenged group on day 1 compared with other tested groups (p < 0.05). However, on days 3 and 5, IL-2 expression decreased in the TM *E. coli-*challenged group compared with the control *E. coli-*challenged group, which increased its IL-2 expression to have the highest level on day 5 among all tested treatment groups (Figure-2) (p < 0.05).

Interleukin-2 is a growth factor that promotes T-cell production. It is mainly produced by naïve T-helper cells, and functions in various immune mechanisms, including responses associated with microbial infections [52]. Interleukin-2 is overexpressed in animal intestines after *E. coli* challenge and viral infections [53, 54]. In this study, IL-2 decreased in the TM *E. coli*-challenged group compared with the control group on day 5. This indicates a long-term effect of TM in improving immune responses, including the dynamic expression of specific cytokines [25]. Moreover, because TM improved tissue stability [42] and oxidative stress tolerance [55], it can positively impact the ability of chickens to resist *E. coli* infection.

The NS-injected control group had approximately the same level of IL-6 expression at all days of post-*E. coli* challenge (Figure-2). However, these levels were lower than those in the TM group, which expressed IL-6 at approximately the same levels on all days of post-*E. coli* challenge. After *E. coli* challenge, the control group expressed significantly higher levels of IL-6 than TM *E. coli-*challenged and NS-injected control groups on days 1 and 3 (p < 0.05). Interleukin-6 expression significantly decreased on day 5, but was higher than that in the TM *E. coli*-challenged group, which recorded the lowest expression on day 5 of post-*E. coli* challenge (p < 0.05). Notably, TM *E. coli*-challenged groups expressed the same level of IL-6 on days 1 and 3, with a remarkable decrease on day 5.

Interleukin-6 is a secreted cytokine that recruits cells involved in innate and acquired immune responses. Interleukin-6 stimulates the production and differentiation of immune cells [43]. It plays a significant role in the proinflammatory response because it helps in short-term protection from infection and damage by activating the immune system. Dysregulation of IL-6 can cause diseases [56]. In this study, the saline-injected groups had lower IL-6 expression than the *E. coli*-challenged groups. Exposure to internal or external stressors was reported to cause IL-6 overexpression. Elnagar *et al*. [43] reported that IL-6 is overexpressed in *E. coli*-challenged broiler chickens. The intestines of *E. coli*-challenged broiler chickens expressed higher levels of proinflammatory cytokines, such as IL-6 [57]. Conversely, IL-6 expression was upregulated in heat-stressed broiler chickens, specifically in the duodenum and jejunum [49].

In this study, IL-6 expression was higher in the saline-injected TM group compared with the control groups; however, the dynamics of IL-6 expression improved in the TM group (Figure-2). Interleukin-6 is crucial in stimulating cells associated with innate and acquired immunity. The results suggest that TM improves the chicken’s immune system to reach homeostasis [25].

The TM *E. coli*-challenged groups recorded lower levels of expression than the *E. coli*-challenged control group on all days of post-*E. coli* challenge. The lower expression level in the TM *E. coli-*challenged group indicates the long-term effect of TM in improving the immune responses, including the dynamics of cytokine expression [25], tissue stability [42], and oxidative stress tolerance [55], which in turn improve the chicken’s ability to resist *E. coli* infection and the subsequent inflammatory response.

The NS control group showed a stable expression level of IL-8 on all days of post-*E. coli* challenge, whereas the TM group reported the lowest expression level on day 5 (Figure-2). However, the expression levels were detected to be higher in the control group than in the TM group on day 5. After the *E. coli* challenge, the IL-8 expression level remarkably increased in the control group on day 3, and then decreased significantly on day 5 (p < 0.05). Thermal manipulation showed a slight decrease in the expression levels on day 3, and then tended to increase expression to the same level as that on day 1 of post-*E. coli* challenge (p > 0.05). Interestingly, the TM group had lower expression levels on all days post-*E. coli* challenge than the control group.

Interleukin-8 is a crucial chemokine in birds. It interacts with the chemokine receptor type 1 (CXCR1) on immune cells to recruit them at the affected site. Interleukin-8 is associated with autoimmune and acute or chronic inflammatory diseases [58]. Studies have investigated the upregulation of IL-8 upon exposure to different stressors. Broiler chickens exposed to the *E. coli* challenge, specifically *E. coli* O78, showed a remarkable increase in the ileal expression of IL-8 [43]. Interleukin-8 overexpression was reported in chicken spleen and cecal tissues after *Eimeria tenella* infection [58]. Thermal manipulation could significantly increase the expression of IL-8 mRNA in the spleen of broiler chicken embryos [24]. In this study, the *E. coli*-infected TM group showed an improved immune response compared with the control group, because TM recorded lower expression levels of IL-8 than the control group.

### Effects of TM and *E. coli* challenge on IL-12, IL-15, and IL-16 expression

**Figure-3:**
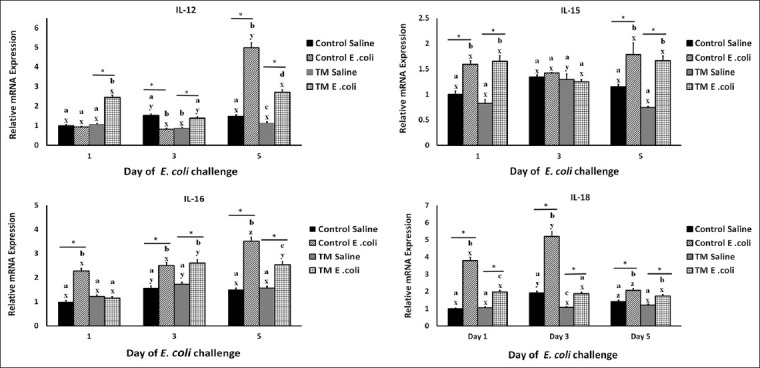
Effects of *Escherichia coli* challenge on the splenic mRNA level of interleukin (IL)-12, IL-15, IL-16, and IL-18 in broiler chickens subjected to thermal manipulation (TM) during embryogenesis (n = 100). The ILs’ mRNA level of control Day-O (saline-injected) is set at 1-fold change, and the levels are presented as fold inductions relative to the control Day-O (saline-injected) group. *Within the same day, the mean of TM group is significantly different compared to the mean of the control (p < 0.05). ^a-b^Within the same day and between different treatment groups, means with different superscripts is significantly different (p < 0.05). ^w-z^Within the same treatment group and between day 0 versus. Days 1, 3, 5, and 7 after *E. coli* challenge, means with different superscripts are significantly different (p < 0.05).

The effects of TM and *E. coli* challenge on IL-12, IL-15, and IL-16 are shown in Figure-3. On day 1, the levels of IL-12 expression in the NS-injected groups were approximately the same. Then, the levels increased on days 3 and 5 in the control group compared with the TM group. After the *E. coli* challenge, the level of IL-12 expression was the same on days 1 and 3 and significantly increased on day 5 (p < 0.05) in the control group. However, the level of IL-12 expression was significantly higher in the TM-treated *E. coli-*challenged group (p < 0.05). Moreover, the TM *E. coli-*challenged group reported the lowest expression level on day 3, with approximately the same expression level on days 1 and 5 of the *E. coli* challenge (Figure-3).

Interleukin-12 is the first identified heterodimeric cytokine. Macrophages and monocyte antigen-presenting cells produce it to activate natural killer cells and naïve CD4+ T lymphocytes. On activation, naïve CD4+ T lymphocytes produce IFN-γ, which stimulates the antigen-presenting cells to increase IL-12 secretion in a positive feedback loop [59].

Studies have reported the upregulation of IL-12 expression on exposure to stress. Salmonella could trigger the differential expression of cytokine genes, including IL-12, in broiler chickens to promote an inflammatory response, which primed an adaptive response [60]. *Eimeria tenella* infection led to the remarkable upregulation of IL-12b expression [61]. Interleukin-12 was overexpressed when broiler chickens were exposed to heat stress and LPS challenge [62].

Consistently, in this study, IL-12 expression increased in the *E. coli*-challenged control group compared with the TM *E. coli*-challenged group on day 5 (Figure-3). The lower expression in the TM *E. coli-*challenged group indicates the long-term effect of TM in improving the immune response, including cytokine expression [25]. The effects of TM in improving tissue stability [42] and oxidative stress tolerance [55] can positively impact the chicken’s ability to resist *E. coli*.

Both saline-injected groups had higher IL-15 expression on day 3, with a slight decrease on day 5 of post-*E. coli* challenge, which was comparable to that recorded on day 1. However, IL-5 expression was lower in the TM group than in the control group on all days of post-*E. coli* challenge (Figure-3).

Post-*E. coli* challenge results showed that IL-15 expression was higher in both *E. coli-*challenged groups than the NS-injected group on day 1. In contrast, on day 3, the TM *E. coli-*challenged group had the lowest level of IL-15 expression among the four tested groups (p < 0.05). On day 5, the control *E. coli-*challenged group had increased expression and recorded a higher level than that detected in the TM *E. coli-*challenged and NS-injected groups (p < 0.05).

In this study, *E. coli* challenge significantly increased IL-15 expression in both the control and TM groups on days 1 and 3 (p < 0.05); however, IL-15 expression decreased in the TM *E. coli*-challenged group on day 5 compared with the *E. coli*-challenged control group, taking in consideration the slight difference in the expression levels between both groups (p > 0.05).

Interleukin-15 has a crucial role in the proliferation and differentiation of immune cells, including T-cells, B-cells, natural killer cells, and intestinal epithelial cells. Interleukin-15 stimulates cytokine production from other cells. To the best of our knowledge, this study is the first to investigate the effects of TM and *E. coli* challenge on IL-15 expression. However, acute heat stress reportedly increased IL-15 levels in both TM and control groups [25]. Thus, the increased expression of IL-15 in the *E. coli-*challenged groups and the slight differences in expression between both may indicate the effect of TM on the immune system of chickens to maintain homeostasis.

The NS-injected groups showed an increase in IL-16 expression on day 3 compared with that on day 1; however, the levels decreased on day 5 and were comparable with those on day 1 (Figure-3). The TM group had a slightly higher level of expression than the control group at all days of the post-*E. coli* challenge. After the *E. coli* challenge, expression gradually increased in the control group and reached the highest level on day 5 (p < 0.05), whereas in the TM group, expression increased on day 3 and remained the same on day 5. However, IL-16 expression was lower in the TM group than in the control group on day 5 (p < 0.05).

Interleukin-16 expression increased slightly in the saline-injected TM group compared with the control group. Interleukin-16 is a proinflammatory cytokine that has a significant role in the innate immune response and the stimulation of the acute phase response [63]. Proinflammatory cytokines have a crucial role in the repair and regeneration of injured tissues [64]. The literature lacks data on the mode of IL-16 expression during exposure to stress conditions. However, the results of this study indicate improved proinflammatory cytokine expression in TM *E. coli*-challenged broiler chickens.

### Effects of TM and *E. coli* challenge on IL-18 and IFN-γ expression

**Figure-4:**
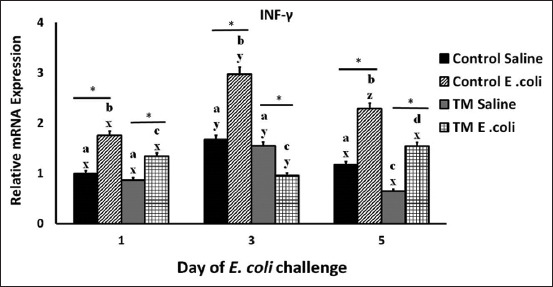
Effects of *Escherichia coli* challenge on the splenic mRNA level of interferon (IFN)-γ in broiler chickens subjected to thermal manipulation (TM) during embryogenesis (n = 100). The IFN-γ mRNA level of control Day-O (saline-injected) is set at 1-fold change, and the levels are presented as fold inductions relative to the control Day-O (saline-injected) group. *Within the same day, the mean of TM group is significantly different compared to the mean of the control (p < 0.05). ^a-b^Within the same day and between different treatment groups, means with different superscripts is significantly different (p < 0.05). ^w-z^Within the same treatment group and between day 0 versus. Days 1, 3, 5, and 7 after *E. coli* challenge, means with different superscripts are significantly different (p < 0.05).

Figure-4 depicts the effect TM and *E. coli* challenge on IL-18 and IFN-γ expression in broiler chickens. The NS TM-injected group showed stable expression of IL-18 on all days of post-*E. coli* challenge, whereas the control group reported the highest expression on day 3. However, the levels of expression were detected to be higher in the control group than in the TM group on days 3 and 5.

After the *E. coli* challenge, IL-18 expression increased remarkably in the control group on day 3 (p < 0.05) and then decreased significantly to the lowest level on day 5 (p < 0.05). The TM group showed a mostly stable expression level on all days of post-*E. coli* challenge (Figure-4). Interestingly, the TM group had a lower expression level on all days of post-*E. coli* challenge compared with the control group, and higher expression levels on all days compared with the NS TM group. In NS-injected groups, the highest expression was recorded on day 3 for both control and TM groups, and the level of IFN-γ expression was detected to be the same on days 1 and 5. However, the TM group had lower expression levels than the control group on all days of the post-*E. coli* challenge (p < 0.05).

After *E. coli* challenge, the *E. coli*-challenged control group recorded a remarkable increase in expression on day 3 (p < 0.05), which was the highest among all days of post-*E. coli* challenge for this group (p < 0.05). The TM *E. coli*-challenged group had decreased expression on day 3 compared with that on day 1. The level increased again and was comparable with that on day 1. However, the levels of IFN-γ expression were lower in the TM *E. coli*-challenged group on all days of post-*E. coli* challenge than the control group (Figure-4) (p < 0.05).

Studies have reported the upregulation of IL-18 and IFN-γ in broiler chickens after *Salmonella* infection [65–68]. Moreover, stress caused by administering corticosterone increased IL-18 expression in broiler chickens [69]. In this study, the *E. coli*-infected TM group showed an improved proinflammatory response compared with the control group, as the TM group recorded lower expression of IL-18 and IFN-γ than the control group.

## Conclusion

In this study, thermally manipulated chicken performance parameters were enhanced compared with the control group during *E. coli* challenge. Body weight was significantly higher in the TM groups than in the control groups. Body temperature was lower in the TM group compared with the control group. The expression level of different genes in the TM group subjected to bacterial infection was more stable than that in the control group subjected to bacterial infection. This study demonstrated that TM (incubation at 39°C air temperature and 65% RH for 18 h daily during ED 10–18) during the second half of the incubation period might modulate the immune system parameters during the secondary post-hatch life and improve the inflammatory response resulting from exposure of chickens to *E. coli* infection.

## Authors’ Contributions

MBA, ZWJ, and MMA: Conceptualization, investigation, data curation, roles/writing–original draft. KMMS, MZO, AA, and MHA: Sample collection and laboratory work supervision, formal analysis, and drafted the manuscript. MBA, ZWJ, and MMA: Manuscript revision. All authors have read, reviewed, and approved the final manuscript.
